# A Rare Case of Acute Cholecystitis Caused by Methicillin-Resistant Staphylococcus aureus (MRSA) in an Immunocompetent Person in the Absence of Bacteremia or Pre-Existing Conditions

**DOI:** 10.7759/cureus.39653

**Published:** 2023-05-29

**Authors:** Ajanta Choudhury, Trupti Pandit, Prabal Chourasia, Ramesh Pandit

**Affiliations:** 1 Internal Medicine, Dhaka Medical College and Hospital, Dhaka, BGD; 2 Pediatrics, Inspira Medical Center, Vineland, USA; 3 Hospital Medicine, Mary Washington Hospital, Fredericksburg, USA; 4 Medicine, Independent Researcher, Philadelphia, USA; 5 Hospital Medicine, University of Pennsylvania / Chester County Hospital, Philadelphia, USA

**Keywords:** gallbladder, mrsa, bacteremia, immunocompetence, acute cholecystitis

## Abstract

Acute cholecystitis, typically caused by gallstone obstruction of the cystic duct, is often complicated by infection. Mostly observed in immunocompromised patients with bacteremia Methicillin-resistant Staphylococcus aureus (MRSA) is not typically associated with this ailment. Here, we present a unique case of acute cholecystitis caused by MRSA in an immunocompetent patient without bacteremia or underlying disease. A male patient aged 59 years was admitted complaining of severe abdominal pain and nausea. Subsequent investigation confirmed acute calculous cholecystitis and thereafter, the patient underwent laparoscopic cholecystectomy. Gallbladder fluid culture indicated elevated quantities of MRSA growths, and suitable antimicrobial therapy was given as part of the treatment process. This exceptional case underlines the significance of recognizing MRSA as a potential pathogen in severe acute cholecystitis cases, particularly those with severe symptoms. Rapid identification and usage of anti-MRSA antibiotics play a crucial role in managing MRSA-related situations. Healthcare providers need to bear in mind the possibility of cholecystitis associated with MRSA particularly when conventional risk factors are not present. Timely intervention is essential for favorable patient outcomes.

## Introduction

Acute cholecystitis is characterized by an inflammation of the gallbladder, often resulting from an obstruction of the cystic duct by gallstones [1]. Infection may complicate 40% to 75% of cases of acute cholecystitis [2]. The most isolated organisms in acute cholecystitis bile cultures are Escherichia coli, Enterococcus, Klebsiella, Clostridium, Bacteroides, and Enterobacter. However, only about 0.8% to 5.6% of organisms cultured from gallbladder specimens include Staphylococcus aureus (S. aureus) [1,3].

Cholecystitis caused by methicillin-resistant Staphylococcus aureus (MRSA) is scarcely reported in the literature. Only 11 cases [1] have been reported, most of which were found in patients with bacteremia or immune dysfunction [2]. Here we present a unique case of acute cholecystitis caused by MRSA in an immunocompetent patient without bacteremia or underlying disease. Hence, in patients with acute onset severe cholecystitis, MRSA testing with gallbladder fluid cultures should be considered. Treatment with anti-MRSA antibiotics needs to be started as soon as possible.

## Case presentation

We present a case of a 59-year-old male with unremarkable medical history except for an episode of provoked pulmonary embolism three years ago, now presented to the hospital with acute onset abdominal pain and nausea about 3 hours before presenting to the emergency department (ED). The pain was noted in the upper abdominal region, radiating around like a band towards the back, dull throbbing in nature, moderate intensity, constant since the gradual onset, associated with nausea, without any aggravating or relieving factors. The patient denied fever, chills, cough, vomiting, constipation, diarrhea, chest pain, or shortness of breath. Social history was notable for never smoking and social alcohol use (average two drinks/week).

Vitals were noted to be: Afebrile, blood pressure (BP): 157/102; heart rate (HR): 89; respiratory rate (RR): 24; SpO2 100% on room air, with body mass index (BMI) of 31.3. His vitals improved to BP 142/85 and RR 11 after pain control in ED. His exam was notable for tenderness to palpation over the right upper quadrant and epigastric region. Lab results were notable for white blood cells (WBC) 14.78 k/µL, hemoglobin (Hb) 16.2 g/dl, neutrophil 79%, lactate dehydrogenase (LDH) 264, carbon dioxide (CO_2_) 18 mmol/L, with calculated anion gap 17, lipase 13 U/L. Abdominal computed tomography (CT) scan findings included a distended gallbladder with cholelithiasis with mild pericholecystic fat stranding, confirming the diagnosis of acute calculous cholecystitis. The patient was started on intravenous (IV) ceftriaxone and metronidazole to cover common enteric pathogens and underwent laparoscopic cholecystectomy. During the procedure, the patient was noted to have a distended and gangrenous appearing gallbladder, with acute inflammation of the gallbladder and surrounding structure. Gallbladder aspiration revealed normal-appearing bile. The bile fluid culture resulted in heavy growth of MRSA, with resistance to Penicillin, Oxacillin; sensitive to clindamycin, doxycycline, and linezolid. During the inpatient stay, the patient’s antibiotics were changed to Clindamycin and Rocephin. Repeat gallbladder area drain culture two days later was negative for any organisms. The patient was tested for nasal MRSA and was negative. Subsequently, the patient was discharged on linezolid for two weeks of antibiotic coverage. All the relevant lab values are provided in Table 1.

**Table 1:** Lab results

Lab result (Reference range)	Day 0	Day 1 (after surgery)	Day 2	Day 4
White Blood Cells (3.5-10 k/µL)	14.78	19.74	9.67	17.42
Hemoglobin (13-17.7 g/dL)	16.2	15	13.2	12.1
Neutrophils (40-75%)	79	83	Not done	68
Monocytes (0.15-1.30 K/µL)	0.88	1.84	Not done	0.38

## Discussion

Methicillin-resistant Staphylococcus aureus (MRSA) has become a major problem worldwide and is associated with significant morbidity and mortality. While S. aureus infections can affect various organ systems, involvement of the biliary tree is exceptionally uncommon [1]. Infection with the gram-positive pathogen S. aureus can lead to a broad range of invasive illnesses, including skin and soft tissue infections, pneumonia, osteomyelitis, septic arthritis, and infective endocarditis [1]. However, it is rarely reported as a biliary pathogen, as the gastrointestinal tract does not harbor S. aureus as normal flora. In a review of 13 case series, including 782 isolates from patients with acute cholecystitis, the frequency of S. aureus was found to be only 2% [1].

In the cases reported previously, patients were found to have staph aureus cholecystitis either from a hematogenous spread of infection to the gallbladder or due to a long-standing intravascular catheter [1]. In contrast, our patient did not have any evidence of metastatic infection or endovascular source. Kim et al. reported a case of MRSA cholecystitis in an HIV patient stating the importance of immunosuppression in developing staph cholecystitis [4].

Our patient is a 59-year-old male who presented with acute onset severe abdominal pain and nausea without any sign of bacteremia or preexisting condition. The patient was started on IV broad spectrum coverage and underwent laparoscopic cholecystectomy. Considering the rising prevalence of community-acquired MRSA globally, bile fluid was sent for MRSA culture, resulting in heavy MRSA growth. The antibiotic was subsequently changed, which led to rapid clinical improvement. In the absence of a clear endovascular source, history of recurrent hospitalization, or recent antibiotic use, we considered the possibility of ascending infection from the duodenal secretions, which was initially swallowed from the nasal discharge [1] since 25-30% of people carry staph bacteria in their noses without being infected by it [3]. Another hypothesis could be the patient already had unidentified bacteremia, as the blood culture was not drawn before the initiation of the empiric antibiotic. Our case is unique, with the gallbladder being the primary focus of MRSA infection. In cases where uncommon presentations of infections occur, potentially leading to severe complications by MRSA, it may be necessary to consider using anti-MRSA antimicrobials, like vancomycin, as part of the initial empirical therapy while awaiting culture results.

Center for Disease Control (CDC) recommends that decolonization regimen could potentially play a role in preventing the recurrence of infection by MRSA [5]. However, further research is required to determine their effectiveness and determine the best regimen to use in community settings. It is advisable to reinforce hygiene practices, provide appropriate wound care, as well as consulting an infectious disease specialist following the treatment of active infection [4].

A comparison of our case with already reported cases in the literature is shown in Table 2.

**Table 2:** Comparison with reported cases in the literature

Reported cases	Age	Underlying comorbidity	Bacteremia
Nepal et al. [1]	Middle Aged	Absent	Present
Gorny [3]	68 years	Type 2 diabetes mellitus with end-stage renal disease on hemodialysis and hypertension	Absent
Kim et al. [4]	36 years	Human Immunodeficiency Virus	Present
Tascini et al. [6]	72 years	Acute exacerbation of chronic obstructive pulmonary disease (COPD) followed by Dissecting abdominal aorta	Present
Martin et al. [7]	Not mentioned	End-stage renal disease	Present
Su and Yu [8]	84 years	End-stage renal disease on hemodialysis	Absent
Nguyen and Ramus [9]	75 years	Post-surgical	Present

## Conclusions

In summary, we report a unique and to our knowledge the first reported case of acute cholecystitis caused primarily by MRSA without bacteremia or history of immune dysfunction or underlying comorbidity. This case expands the broad range of serious infections that MRSA can cause. Physicians should be aware that S. aureus, including MRSA, can cause acute cholecystitis and should be considered in the differential diagnosis. Appropriate, timely intervention and proper antibiotics can prevent adverse patient outcomes.
